# Development of a computational strategy to compare repetitive element enrichment between experimental conditions from high-throughput sequencing datasets

**DOI:** 10.1186/1753-6561-6-S6-O29

**Published:** 2012-10-01

**Authors:** Steven Criscione, Nicola Neretti

**Affiliations:** 1 Molecular Biology, Cell Biology, and Biochemistry, Brown University, 75 Waterman Street, Providence, RI 02912, USA; 2 Center for Computational Molecular Biology, and Biochemistry, Brown University, Providence, RI 02912, USA

## 

Repetitive and transposable elements comprise more than half of the human genome and play diverse roles in many biological processes. Mobile elements including retrotransposons are implicated in the organization of the epigenetic landscape, the progression of tumorigenesis, and the enhancement of genetic diversity. Despite the importance of repetitive and transposable elements these sequences are traditionally ignored in high-throughput sequencing analysis due to the technical difficulty of uniquely mapping reads from repeat DNA sequences. Here we report a new computational method for the analysis of repetitive elements from high-throughput sequencing datasets that accounts for all mapping reads. In our approach, we examine reads that map uniquely and to multiple locations of the genome using two separate strategies to determine a complete estimate of enrichment for repetitive elements. Included in our computational method is an output defined by reads per kilobase of repeat element per million mapped reads (similar to RPKM definition for the exon model) [1]. The calculated repeat element enrichment RPKM allows for the comparisons between repetitive elements as well as between experimental conditions. Our new method for examining repetitive elements from high-throughput sequencing datasets represents an improvement over existing methods because we do not exclude reads from the analysis and we can make comparisons between experimental conditions. To test our method we have examined repetitive element enrichment in the embryonic and adult mouse across different tissues using a variety of high-throughput mouse sequencing datasets available from the mouse ENCODE project and Shen *et al.* that provide a thorough snapshot of the epigenetic landscape of the embryonic and adult mouse [2]. We compare our method with an existing strategy for estimating repetitive element enrichment proposed by Day *et al. *[3], and demonstrate the advantages to our strategy. In addition, we test the robustness of our approach for determining differences in enrichment between experimental samples by conducting a comparison between the embryonic and adult mouse.
